# Applicator volume elimination methods and their influence on DVH analysis of HDR prostate implants

**DOI:** 10.1120/jacmp.v7i2.2213

**Published:** 2006-05-25

**Authors:** Nagarajan Vivekanandan, Bhuvana Kunjithapatham, Nithya Kanakavelu, Balakrishnan Shankarrao Irde, Lakshmanan Alathurmadam Vaidyanathan

**Affiliations:** ^1^ Department of Medical Physics Cancer Institute Adyar Chennai 600 020 India

**Keywords:** brachytherapy, dose‐volume analysis

## Abstract

In CT anatomy‐based inverse treatment planning of interstitial high‐dose rate (HDR) brachytherapy planning, the planning target volume (PTV) delineated by the radiation oncologist includes the applicator volume. The applicator volume can be eliminated with the help of two methods: one based on Boolean operations and the other using the erasing option of the application software. Both methods are compared, and the results are discussed. Elimination of the applicator volume results in the proper estimation of the PTV and the exclusion of the clinically insignificant hot volume from the PTV in the dose‐volume histogram (DVH) analysis. Five cases of prostate cancer are considered for analysis. The PTV, applicator volume, maximum, mean, modal, and minimum dose, and the percentage volume of the PTV structure receiving the percent dose for both cases, that is, with and without applicator volume, are tabulated and analyzed. The elimination of the applicator volume results in the proper volume estimation of the PTV structure and leads to better DVH analysis of interstitial HDR prostate implants. The procedure would have little relevance in routine planning but improves dose reporting. It is too early to conclude its clinical significance or insignificance.

PACS numbers: 57.53.Jw, 87.53.Tf

## I. INTRODUCTION

Inverse planning is an optimization process adapted to the individual geometry of the patient. The inverse planning algorithm is anatomy‐based and considers the real anatomy of the tumor and the organs at risk (OARs). This anatomy‐based optimization^(^ ^1^ ^,^ ^2^ ^)^ enables the automatic calculation of dwell times to give the desired dose to selected structures. The dose constraints are specified for the selected structure by setting an objective dose toward which the dose calculation iterates, plus the maximum deviation from the objective. Tumor dose is specified by setting constraints in the dose‐volume histogram (DVH) itself. The optimization algorithm then tries to find the optimal dwell times that meet these constraints. Inverse planning provides solutions that protect the OARs and the normal tissues better than empirical methods. With this optimization, it is possible to obtain the optimal number of catheters, their position, and the optimum distribution of dwell times in high‐dose rate (HDR) brachytherapy.

In this study on interstitial HDR brachytherapy planning of prostate, first the clinical target volume was delineated, that is, the volume of prostate tissue that has to be treated adequately. The planning target volume (PTV) is identical to the clinical target volume because no margins are required due to consistency of source positions.^(3)^ The OARs rectum and urethra were also delineated. The prescribed dose is either 4800 cGy in two courses of 2400 cGy each, that is, four fractions of 600 cGy each, or external beam therapy followed by a boost dose of 1950 cGy in three fractions of 650 cGy each. The dose constraints to the PTV are set as at least 100% volume to receive 100% of the prescribed dose as the lower limit and 10% volume not to receive more than 300% of the prescribed dose as the upper limit. The upper dose constraints for the urethra are set as 120% and for the rectum as 75%.

The delineated PTV includes the applicator volume, which leads to improper volume estimation of the PTV structure and DVH analysis. Two methods are proposed to eliminate the applicator volume from the PTV in this work. The first method is based on the use of Boolean operators in the DVH module. In addition to the PTV, the applicator volume is created during the contouring process. Using a logic operator (XOR), the applicator volume is then eliminated from the PTV in the DVH analysis. The other method uses the simple erase tool. A copy of the PTV is first created and the applicator area is removed from the PTV in each CT slice‐using eraser. Both methods are compared and analyzed.

## II. MATERIALS AND METHODS

### A. Elimination of applicator volume by using “logic” or “Boolean operators”

**Figure 1:**
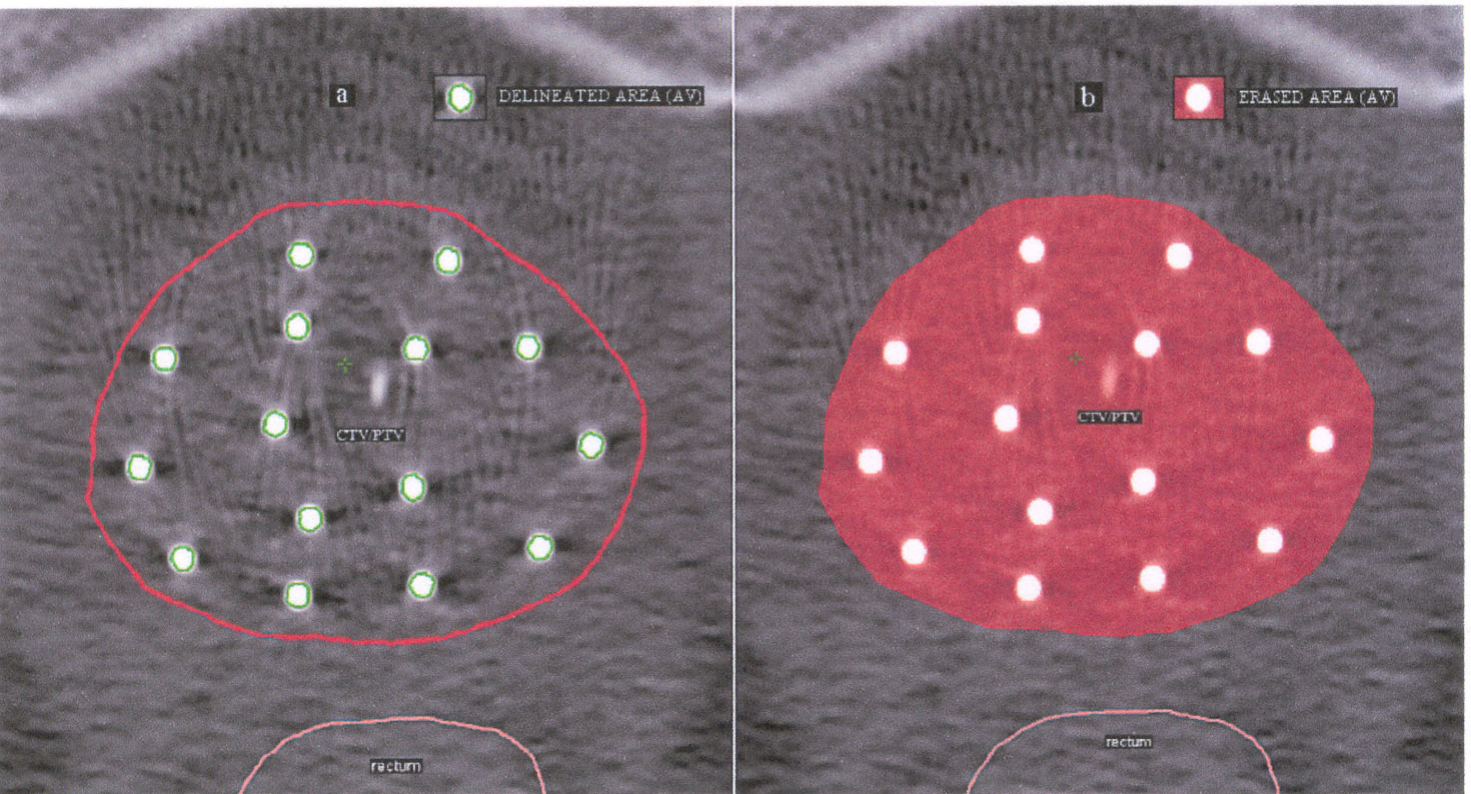
(a). Delineation of PTV and definition of applicator volume. Two structures, PTV (planning target volume) and AV (applicator volume), are created separately using the contouring tools of the application software. CTV is the clinical target volume. (b) Elimination of applicator volume using the erase tool. The applicator area within the PTV structure is carefully erased and renamed PTV‐AV.

In CT anatomy‐based inverse treatment planning of HDR prostate brachytherapy, after the delineation of the PTV and the definition of OARs, a new volume is defined inside the PTV structure, namely, the applicator volume. This is shown in Fig. 1(a). All the applicator surfaces are contoured either manually or automatically using the segmentation wizard of the brachyvision module of the ECLIPSE treatment‐planning system. The area of the applicators determined in axial images agrees well with the actual calculated physical area of the needles. The volume of the applicators measured is also verified with the calculated applicator volume. In this study, a deviation of 2% to 3% was noted between calculated and measured values. Care must be taken to avoid excessive contouring of the applicators beyond PTV structures. This problem is often noticed if the applicator volume is delineated automatically using software options. Erasing or removing the applicator volume beyond the PTV using post‐processing tools can easily solve this problem. The dose constraints were specified for the PTV and the OARs, and volume optimization or inverse planning was carried out for all five patients considered in this study.

**Figure 2:**
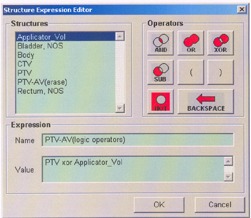
Structure‐expression editor of the DVH module. In the structure‐expression editor, an expression is named PTV‐AV (planning target volume without applicator volume) and the value PTV XOR AV.

**Figure 3:**
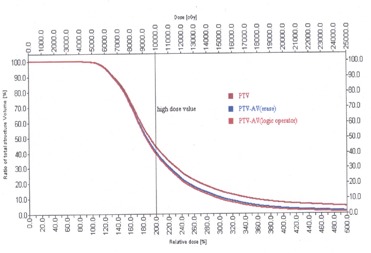
The cumulative dose‐volume histogram for PTV with and without the applicator volume by the Boolean operator method and the erase tool method.

Using the structure‐expression editor of the DVH module, the applicator volume can be eliminated in the DVH analysis.^(4)^ The expression is named PTV‐AV. The elimination is achieved by using a suitable logic value (PTV XOR AV). This is illustrated in Fig. 2. The cumulative DVH for the PTV and the PTV without applicator volume is shown in Fig. 3. From the dose‐volume histograms, the PTV, maximum, mean, modal, and median dose (Table 1, and the percentage volume of the PTV structure receiving the percent dose for all five prostate cases are tabulated (Table 2.

**Table 1:** Volume and dose data for the PTV structure with and without the applicator volume

Patient no.	Dose / fraction	Structure	Volume $\left({\text{cm}}^{3}\right)$	% Vol change	Max % dose	Mean % dose	Modal % dose	Median % dose	STD
1	600	PTV	59.8	9.4	3439.7	281.2	139.7	224.8	204.06
		PTV‐AV	54.2		2625.0	278.4	139.7	221.8	171.21
2	650	PTV	75.3	5.2	2315.7	202.5	148.1	180.5	104.34
		PTV‐AV	71.4		1525.9	191.3	148.1	178.6	76.41
3	650	PTV	34.9	11.5	2756.3	232.3	242.4	190.6	154.46
		PTV‐AV	30.9		1719.4	229.3	242.4	187.1	101.01
4	600	PTV	61.3	6.7	2626.2	225.1	212.1	197.2	150.26
		PTV‐AV	57.2		1625.1	199.2	212.1	195.8	121.08
5	600	PTV	57.1	7.2	2862.1	240.2	181.3	230.2	186.26
		PTV‐AV	53.0		1842.1	229.1	181.3	227.0	134.55

**Table 2:** Percent volume of the PTV receiving the percent dose with and without the applicator volume

% Dose	Patient 1		Patient 2		Patient 3		Patient 4		Patient 5	
	PTV % vol.	PTV‐AV % vol.	PTV % vol.	PTV‐AV % vol.	PTV % vol.	PTV‐AV % vol.	PTV % vol.	PTV‐AV % vol.	PTV % vol.	PTV‐AV % vol.
100	100	100	99.81	99.79	99.50	99.37	99.85	99.71	99.32	99.11
150	89.06	88.63	77.93	77.55	81.58	81.21	85.26	85.11	89.11	88.94
200	62.39	60.82	33.98	31.22	44.03	41.06	60.39	58.88	46.81	45.87
250	40.91	39.47	14.66	11.80	24.33	20.59	38.11	35.62	35.13	31.99
300	27.70	26.11	8.11	5.62	14.64	11.21	25.21	22.42	11.24	8.10
350	18.56	16.87	4.87	2.85	9.61	6.70	18.23	14.88	8.35	6.06
400	13.27	11.51	3.15	1.22	6.96	3.97	10.92	7.21	4.21	1.33

### B. Elimination of applicator volume using the “erase” option

The applicator volume in the PTV structure can also be eliminated by simply using the “erase” tool. After the delineation of the PTV and the definition of the OARs by the oncologist, the PTV structure is copied and renamed PTV‐AV. Then the eraser tool is chosen, and the smallest possible brush size is selected for the maximum possible accuracy (0.1 cm diameter). It is easier to erase the applicator volume if the CT image is magnified. This is shown in Fig. 1(b). The brachyvision module (version 7.3.10) of the ECLIPSE treatment‐planning system was used for the entire study. The cumulative DVH is illustrated in Fig. 3.

### C. Comparison of the applicator volume elimination methods (Boolean and erase)

For all the patient data, the applicator volume was eliminated by both the Boolean and erase methods, and the cumulative DVH analysis was done. Both methods yield almost the same results. From Fig. 3, it is obvious that both curves coincide such that it is difficult to differentiate the results between the two methods. However, a deviation can be noted in some cases in the high‐dose region.

## III. RESULTS AND DISCUSSION

The delineation of the PTV in CT anatomy‐based HDR brachytherapy treatment planning includes the applicator volume generally. The applicator volume can be eliminated with any one of the two proposed methods. The results are tabulated for five prostate cases. The data listed in Table 1 indicate that the estimated volume of the PTV structure is reduced by 5.2% to 11.5%. There is no change in the modal dose, but the maximum dose drops by a wide margin. The mean dose is reduced by almost 10%. The medial dose varies by 3% to 5% only. The STD also varies by as high as 20%. The data listed in Table 2 indicate the least variation in percentage volume of the PTV structure receiving the percent dose with and without applicator volume in the 0% to 150% dose range. It can be easily explained as follows: this dose region is away from the applicators. In the dose region above 150%, the effect is increasingly greater due to the fact that dose region is either close to or within the applicator.

## IV. CONCLUSION

It can be concluded that the elimination of the applicator volume from the PTV structure results in the proper estimation of the PTV. Both applicator volume elimination methods discussed yield the same results. Of the two methods, the Boolean method with automatic contouring of the applicator volume is simple to practice. The measured and calculated values of the applicator volume show an error of 3% (maximum). The error is due to the limitation of the brush's (circular) diameter or the width of the contour or pixel size. The results indicate a reduction in reported PTV values of as high as 11.5%. The reduced volume does affect the high dose values, as would be expected, since these conform to the catheters, which results in variation in the reported maximum, mean, and medial dose except minimum and modal dose.

By the suggested methods the clinically irrelevant and insignificant hot volume can be excluded from the DVH analysis. As the minimum dose and modal dose fall in the prostate, the elimination of the applicator volume will not result in any difference in treatment planning or assessment. The high‐dose volume values make a difference when they fall in structures such as the urethra, but that volume would not be affected since catheters generally do not pass through the urethra. In summary, the procedure would have little relevance in routine planning but improves dose reporting. It is too early to conclude its clinical significance or insignificance.
